# Alcohol use and support receipt among people experiencing homelessness in England: a cross-sectional analysis with comparison to private household populations

**DOI:** 10.1136/bmjph-2026-004924

**Published:** 2026-08-24

**Authors:** Vera Helen Buss, Sharon Cox, Melissa Oldham, Harry Tattan-Birch, Sarah E Jackson, Debra Hertzberg, Lion Shahab, Jamie Brown, Dimitra Kale

**Affiliations:** 1 Department of Behavioural Science and Health, University College London, London, UK; 2 Behavioural Research UK, Edinburgh, UK; 3 Homeless Link, London, UK

**Keywords:** Cross-Sectional Studies, Prevalence, Public Health, Ill-Housed Persons

## Abstract

**Introduction:**

Alcohol-related harm is more prevalent among people experiencing homelessness, but national data often overlook this group. This study examined alcohol use and receipt of support and compared this group with the general population.

**Methods:**

We analysed data from the Homeless Health Needs Audit (2015–2021; cross-sectional survey of a convenience sample of people experiencing homelessness in England), and for general population estimates, from the nationally representative Health Survey for England (2022). Outcomes included any alcohol use, exceeding drinking guidelines (>14 units per week), possible alcohol use disorder (men: >50 units per week; women: >35 units per week), self-identified alcohol problem and receipt of support for their drinking. We estimated the prevalence for each of the outcomes and odds of receiving support and type of support by age, gender and type of homelessness.

**Results:**

Among people experiencing homelessness (n=2652), 78.4% (95% CI 76.5% to 80.2%) consumed alcohol, 31.1% (95% CI 29.3% to 33.3%) exceeded drinking guidelines and 19.2% (95% CI 17.4% to 20.9%) had possible alcohol use disorder, compared with 83.4% (95% CI 82.4% to 84.4%), 23.9% (95% CI 22.7% to 25.1%) and 4.8% (95% CI 4.2% to 5.5%) in the general population, respectively. For the latter two outcomes, prevalence was highest among people sleeping rough (47.4% (95% CI 40.8% to 54.1) and 37.8% (95% CI 31.4% to 44.2%), respectively). Overall, 28.3% (95% CI 26.5% to 30.0%) self-identified ever having an alcohol problem; of these, 58.4% (95% CI 54.7% to 62.1%) received support. People sleeping rough had lower odds of receiving support than those in their own tenancy at risk of homelessness (OR adjusted for age and gender: 0.26 (95% CI 0.12 to 0.54)).

**Conclusions:**

Similar proportions of people experiencing homelessness and those in private households reported drinking alcohol in the past year. However, people experiencing homelessness were more likely to exceed drinking guidelines and had approximately fourfold higher prevalence of alcohol use disorder. Although nearly two in three individuals self-identifying as having had an alcohol problem received support, access was unequal, with substantially lower odds of support among people sleeping rough.

**WHAT IS ALREADY KNOWN ON THIS TOPIC**

- Homelessness is changing and increasing in England. People experiencing homelessness have higher rates of alcohol use disorder than the general population. However, up-to-date prevalence estimates and information on support people receive are limited.

**WHAT THIS STUDY ADDS**

- People experiencing homelessness had 30% higher rates of exceeding guideline recommendations and 300% higher rates of having possible alcohol use disorder compared with the general population living in private households. The burden of alcohol harms and support needs among people experiencing homelessness is unevenly distributed, with those sleeping rough showing the greatest unmet need.

**HOW THIS STUDY MIGHT AFFECT RESEARCH, PRACTICE OR POLICY**

- People experiencing homelessness who have alcohol use disorders are more likely to drink at high levels and less likely to receive support. Without addressing these inequities, existing health disparities could worsen.

## Introduction

 Homelessness is rising in England, driven by lack of social and affordable housing and generally high living costs.^1^ Rough sleeping—that is, living outdoors or in places not meant for habitation—is the most visible and unsafe form of homelessness.^2^ However, homelessness also includes statutory (eg, accommodation provided by a local council) and hidden forms (eg, sleeping on somebody’s sofa).^1^ Risky alcohol use and dependence are common among people experiencing homelessness, contributing to poor health and inequalities.^3^

Accurate counts are difficult due to varied forms of homelessness and limitations in data collection (eg, street counts miss hidden populations; household surveys exclude non-traditional living arrangements).^4^ From the existing data, the UK-based charity Shelter estimated that in 2024 over 350 000 people, including adults and children, were homeless in England on a single night—about 1 in 160 people.^5^ Of these, the vast majority (~90%) were living in temporary accommodation provided by councils, and around 1% were sleeping rough.^5^ Beyond these numbers, there is limited data on health needs, including for alcohol treatment. As population surveys often exclude people experiencing homelessness, prevalence of health behaviours such as harmful alcohol use may be underestimated, making it harder to allocate resources effectively.

Evidence shows high rates of alcohol use disorder (AUD; encompassing harmful drinking and alcohol dependence)^6^ in people experiencing homelessness. A meta-analysis found a 26% current and 46% lifetime AUD prevalence.^7^ In a specialist homeless primary healthcare centre in England, 21% of the patients had alcohol dependence^3^, which is far higher than in the general population (less than 2%).^8^ Among a sample of people sleeping rough in London in 2000, 68% used alcohol in the past month, with 25% meeting criteria for alcohol dependence.^9^ In 2021, according to official statistics, alcohol accounted for 10% of deaths among people experiencing homelessness in England and Wales^10^ compared with 1% in the general population.^11^ However, national data remains scarce, limiting service planning.

Differences in harmful drinking and treatment access may vary across forms of homelessness, as well as by age and gender, because these factors shape both vulnerability and support opportunities. Individuals in supported housing may have better service access than others, though not guaranteed. Gender and age add further complexity: women often navigate unique challenges,^12^ and older adults may be better informed about available services due to longer periods of homelessness and alcohol dependence, but they may also be more disillusioned due to negative experiences.

Therefore, this study aimed to estimate prevalence of alcohol use, exceeding the UK Chief Medical Officers’ low risk drinking guideline recommendations (hazardous drinking; ie, more than 14 units a week),^13,14^ and possible AUD (harmful drinking or alcohol dependence, ie, 35 or more units for women and 50 or more for men)^14^ among people experiencing homelessness in England and use these figures to reassess national prevalence figures by including this population. Further, the study explored access to alcohol treatment among people experiencing homelessness who self-identified as having a current alcohol problem or being in recovery.

Among people experiencing homelessness, we sought to assess (i) prevalence of alcohol use, exceeding guideline recommendations and possible AUD, (ii) self-identified alcohol problems and related treatment support, (iii) type of support received and (iv) update overall population alcohol use prevalence including data from people experiencing homelessness. Analyses (i–iii) were also stratified by age, gender and type of homelessness. The specific research questions are provided in the study protocol (https://osf.io/r7485/).

## Materials and methods

### Study design and participants

We used data from the Homeless Health Needs Audit (HHNA) survey,^15^ and for general population estimates, from the Health Survey for England (HSE).^16^ The HHNA, a cross-sectional survey, captures the health needs of people experiencing homelessness in local areas of England and assesses their health, lifestyle and use of healthcare services. It was developed in partnership between the charity Homeless Link, the National Health Service, local authorities and homelessness service providers. The HHNA survey includes individuals aged ≥15 years. Between 2015 and 2021, 43 audits including 5916 individuals were completed of whom 2792 (2270 between 2015 and 2017 and 522 between 2018 and 2021) provided consent for their data to be used for research purposes (see^15^ for further information). For this study, we only included adults aged ≥18.

The HSE, a yearly cross-sectional survey, assesses the health of people living in private households in England (ie, it does not include people experiencing homelessness).^16^ It includes participants aged ≥16 years. For this study, we used publicly available data of adults ≥18 from the 2022 survey.

We used the most recent available HSE data (2022), as data collection and representativeness were affected in 2021 due to the COVID-19 pandemic.^17^ Additionally, temporary policy measures during 2020–2021 (eg, the ‘Everyone In’ initiative) altered patterns of rough sleeping^1^, making 2022 a more representative comparison year for estimates of the number of people experiencing homelessness (based on data from the Homelessness Monitor^18^). We did not expect substantial year-to-year variation in alcohol consumption by homelessness type that would materially affect our conclusions. In an unplanned sensitivity analysis, we used 2018 data for the HSE and Homelessness Monitor to derive estimates of household prevalence and the number of people experiencing homelessness.

The research team only had access to the anonymised data. The manuscript follows the Strengthening the Reporting of Observational Studies in Epidemiology (STROBE) statement.^19^ The study protocol was pre-registered on the Open Science Framework (https://osf.io/r7485/).

### Outcome measures and covariates

#### Alcohol use

In HHNA, alcohol use was measured with: ‘How often have you had an alcoholic drink during the last 12 months?’

- Almost every day (scored 6.5)
- 5–6 days per week (5.5)
- 3–4 days per week (3.5)
- 1–2 days per week (1.5)
- 1–2 times per month (0.5).
- Once every couple of months (0.1)
- 1–2 times per year (0.05).
- Not at all (0).
- No answer.

Options (a–g)=drinking alcohol; (h)=no alcohol in past year; (i) missing unless subsequent question confirmed drinking.

In HSE, participants were asked: ‘Do you ever drink alcohol nowadays, including drinks you brew or make at home?’ Those responding ‘yes’ or ‘very occasionally’ (from follow-up question) were classified as drinking.

#### Exceeding guideline recommendations

For HHNA, those reporting drinking were asked: ‘How many units of alcohol do you drink on a typical day when drinking? Please refer to the flashcard to work this out’ (open response). Weekly consumption=units per drinking day×drinking days per week (score from previous question). Those abstaining were assigned zero.

In HSE, weekly consumption was derived from frequency and amount of different drinks over 12 months.^20^ Participants were categorised as exceeding/not exceeding recommended limits of 14 units per week.^13^

#### Possible AUD

Classified as >50 units per week for males or >35 units per week for females, per National Institute for Health and Care Excellence guidelines.^14^

#### Self-identified alcohol problem

HHNA survey: ‘Do you have or are you recovering from an alcohol problem?’ (a) Currently have; (b) In recovery; (c) Yes; (d) No; (e) No answer. (a–c)=ever had an alcohol problem, with separate reporting for (a) and (b); (d)=never; (e) missing.

#### Receiving support

Only asked among those self-identifying an alcohol problem: ‘If you have, or are in recovery from, an alcohol problem, are you receiving support or treatment to help you? Please tick only one’. (a) Yes, meets needs; (b) Yes, but need more help; (c) No, but would help; (d) No, do not need. (a–b)=receiving support; (c–d)=not receiving support (also reported separately).

#### Type of support received

HHNA asked: ‘What support are you receiving to help you address your alcohol use?’

1. Advice/information (eg, general practitioner, accident & emergency department).
2. Mutual aid (eg, Alcoholics Anonymous).
3. Community prescribing (drug treatment prescribed as part of care plan).
4. Counselling/psychological support.
5. Day programmes in community.
6. Detox (inpatient).
7. Residential rehabilitation.
8. Aftercare (support following structured treatment).
9. Peer support.
10. Other.

Options were not mutually exclusive. For regression, we grouped: information/peer-led support (a, b, i); psychological/community-based interventions (d, e, h); medical/residential treatment (c, f, g); other (j).

#### Socio-demographic characteristics

Variables included age (18–24, 25–34, 35–44, 45–54, ≥55 years; for type of support: 18–34 or ≥35), gender (female or male; those identifying otherwise were excluded due to small numbers affecting imputation) and type/history of homelessness.

For prevalence, self-identified problem and receipt of support, type of homelessness was grouped into: housed—own tenancy; hostel/supported accommodation; bed & breakfast/other temporary accommodation; emergency accommodation; rough sleeping; sofa surfing; other. For type of support: housed—own tenancy; temporary/supported housing; unsheltered/hidden homelessness; other. For population estimates: rough sleeping; hostels; unsuitable temporary accommodation (incl. B&B, private non-self-contained licensed/nightly let and out of area placements); sofa surfing; unconventional accommodation (eg, cars, vans, lorries, caravans/motor homes, tents, boats, sheds, garages, industrial/commercial premises). ‘Housed – own tenancy’ refers to people supported by homelessness support services having very recently moved into a property.^21^ Details are in the supplement and protocol.

History of homelessness (‘Have you ever (in your lifetime) done any of the following?’): stayed at hostel/foyer/refuge/night shelter/bed & breakfast/other homelessness service; sofa surfed; slept rough; applied to council as homeless; none. We combined these into one variable indicating the number of different homelessness types experienced. More details are in the online supplemental file 1.

### Analysis

Analyses were conducted in RStudio (V.2022.07.1) using R (V.4.2.1). Missing data were imputed using the mice package (20 imputations),^22^ analysed separately and combined using Rubin’s rule.^23^ Missing data details are in online supplemental tables S1 and S2. We also conducted a complete case sensitivity analysis.

#### Prevalence overall and stratified

Using HHNA data, we estimated prevalence and 95% CIs of alcohol use, exceeding guidelines and possible AUD overall and stratified by age, gender and type of homelessness (raw and adjusted for the other two variables). Additionally, we explored associations between homelessness history and exceeding guidelines or possible AUD, respectively, using logistic regression.

#### Self-identified problem and support

We estimated prevalence (95% CI) of self-identified alcohol problem, and among this group, the proportion (95% CI) receiving support (overall and stratified by age, gender and type of homelessness). Unadjusted and adjusted logistic regression examined associations between receiving support and age, gender, homelessness type and number of homelessness types. Additional analyses distinguished between current versus past alcohol problems and explored unmet support needs and satisfaction.

#### Type of support

We examined variations in support type across socio-demographic groups using descriptive statistics and logistic regression (unadjusted and adjusted). Unplanned analyses considered recovery status and satisfaction with level of support as predictors.

#### Population estimates

We weighted HHNA prevalence estimates by national homelessness proportions (from Homelessness Monitor)^18^ to reassess national estimates of alcohol use, exceeding guidelines and possible AUD among adults in England based on HSE prevalence figures. To retrieve weighted HHNA prevalence estimates, we weighed each subgroup’s prevalence by its population size and then normalised by the total population of people experiencing homelessness. The national prevalence was calculated as a weighted average of the two groups (ie, people experiencing homelessness based on HHNA data and those in private households based on HSE data), where each group’s prevalence was weighted by its population size and then divided by the total population. In an unplanned analysis, we used data from the 2018 editions of the HSE and Homelessness Monitor to derive estimates of household prevalence and the number of people experiencing homelessness. Further details are in the online supplemental file 1. Another unplanned analysis compared mean weekly alcohol consumption in the HHNA to the HSE.

## Results

Most participants (N=2625) were under 55 years of age, male and stayed in a hostel or supported accommodation and had experienced various forms of homelessness (table 1).

**Table 1:** Sociodemographic characteristics of participants (N=2652)

Characteristic	% (95% CI)
Age	
18−24 years	21.8 (20.3 to 23.4)
25−34 years	22.7 (21.1 to 24.3)
35−44 years	22.1 (20.5 to 23.7)
45−54 years	21.8 (20.2 to 23.3)
≥55 years	11.6 (10.4 to 12.8)
Gender*	
Female	27.2 (25.5 to 28.9)
Male	72.8 (71.1 to 74.5)
Type of homelessness	
Housed—own tenancy†	9.0 (7.9 to 10.1)
Hostel or supported accommodation	58.6 (56.7 to 60.5)
Bed & breakfast or other temporary accommodation	5.1 (4.2 to 5.9)
Emergency accommodation	7.5 (6.5 to 8.5)
Rough sleeping	9.4 (8.3 to 10.5)
Sofa surfing	7.2 (6.2 to 8.2)
Other	3.2 (2.5 to 3.8)
Number of types of homelessness experienced‡	
0	2.5 (1.9 to 3.1)
1	12.3 (11.1 to 13.6)
2	18.2 (16.7 to 19.7)
3	27.8 (26.1 to 29.6)
4	39.1 (37.2 to 41.1)

* Exclude due to small numbers: genderqueer n=1, non-binary n=3, transgender n=7, other n=3.

† People supported by homelessness support services having very recently moved into a property.

‡ Options included: stayed at a hostel, foyer, refuge, night shelter or bed & breakfast hotel or any other type of homelessness service; sofa surfed; slept rough; applied to the council as homeless; none of the above.

### Prevalence overall and stratified

Overall, 78.4% of the sample consumed alcohol, 31.1% exceeded guidelines and 19.2% had possible AUD (table 2). Exceeding guidelines and possible AUD were less common in the youngest age group, among females and those with their own tenancy. In contrast, those sleeping rough had particularly high prevalence of exceeding guidelines and possible AUD. Adjusted prevalence estimates are presented in online supplemental table S3. Notably, gender differences became less pronounced after adjusting for age and type of homelessness. For example, the raw AUD prevalence was 15.6% among females and 20.5% for males, compared with adjusted estimates of 17.3% and 19.2%, respectively. This attenuation is largely explained by differences in homelessness type, particularly with females less frequently experiencing rough sleeping than males (4.8% vs 11.1%; see online supplemental table S4).

**Table 2:** Raw prevalence of any alcohol use, exceeding guideline recommendations and possible alcohol overall (N=2652) and among subgroups

Characteristic	Raw prevalence, % (95% CI)		
	Any alcohol use	Exceeding guideline recommendations	Possible alcohol use disorder
Overall	78.4 (76.5 to 80.2)	31.3 (29.3 to 33.3)	19.2 (17.4 to 20.9)
Age			
18–24 years	81.0 (77.7 to 84.3)	19.7 (16.3 to 23.1)	8.6 (6.1 to 11.0)
25–34 years	80.5 (77.1 to 83.8)	32.4 (28.5 to 36.3)	18.4 (15.2 to 21.6)
35–44 years	79.5 (76.0 to 83.0)	35.5 (31.1 to 39.8)	24.8 (21.0 to 28.5)
45–54 years	77.6 (73.5 to 81.7)	36.3 (32.1 to 40.4)	23.3 (19.6 to 27.1)
≥55 years	68.5 (62.8 to 74.1)	33.4 (27.8 to 39.0)	22.1 (17.1 to 27.1)
Gender			
Female	76.2 (72.9 to 79.5)	23.7 (20.5 to 26.9)	15.6 (12.8 to 18.4)
Male	79.2 (77.0 to 81.3)	34.1 (31.7 to 36.5)	20.5 (18.4 to 22.6)
Type of homelessness			
Housed—own tenancy*	73.3 (67.5 to 79.2)	23.6 (18.1 to 29.1)	13.1 (8.7 to 17.5)
Hostel or supported accommodation	79.5 (77.2 to 81.8)	30.1 (27.6 to 32.6)	17.1 (15.0 to 19.1)
Bed & breakfast or other temporary accommodation	72.3 (64.3 to 80.2)	24.5 (16.6 to 32.4)	16.4 (9.5 to 23.3)
Emergency accommodation	77.9 (71.3 to 84.6)	28.3 (21.3 to 35.6)	18.2 (12.3 to 24.2)
Rough sleeping	81.6 (76.4 to 86.7)	47.4 (40.8 to 54.1)	37.8 (31.4 to 44.2)
Sofa surfing	79.5 (73.4 to 85.6)	38.2 (31.1 to 45.4)	23.5 (17.2 to 29.8)
Other	69.9 (59.0 to 80.9)	28.2 (17.8 to 38.5)	16.9 (8.3 to 25.4)

* People supported by homelessness support services having very recently moved into a property.

The odds of drinking above guideline recommendations increased with the number of different types of homelessness experienced (OR=1.32 (95% CI 1.22 to 1.43); adjusted for age and gender (OR_adj_)=1.31 (95% CI 1.21 to 1.41)). A similar pattern was observed for possible AUD (OR=1.45 (95% CI 1.32 to 1.60), OR_adj_=1.45 (95% CI 1.31 to 1.60)).

### Self-identified problem and support

Overall, 28.3% of participants self-identified as having had an alcohol problem, and among them, 58.4% reported receiving support (table 3). Self-identification of an alcohol problem was less common among the youngest age group (7.6%) and among females (22.6%), which is in line with AUD prevalence estimates (table 2). Individuals who slept rough had significantly lower odds of receiving support than those in their own tenancy (OR_adj_=0.26 (95% CI 0.12 to 0.54)). There were no further statistically significant differences, likely due to limited statistical power. However, there was some indication that males may have lower odds of receiving support than females (OR_adj_=0.72 (95% CI 0.49 to 1.07)). There was a negative association between having received support and the number of homelessness types experienced (OR_adj_=0.86 (95% CI 0.74 to 1.00)).

**Table 3:** Percentage of people self-identifying ever having had an alcohol problem (N=2652), and among these (n=749), percentage who have received support for it overall and among subgroups, as well as unadjusted and adjusted ORs for receiving support

Characteristic	Self-identified alcohol problem	Receipt of support		
	% (95% CI)	% (95% CI)	OR (95% CI)	OR_adj_ (95% CI)
Overall	28.3 (26.5 to 30.0)	58.4 (54.7 to 62.1)	‒	‒
Age				
18–24 years	7.6 (5.4 to 9.9)	47.3 (32.1 to 62.4)	0.67 (0.33 to 1.37)	0.71 (0.33 to 1.52)
25–34 years	28.1 (24.4 to 31.9)	51.3 (43.4 to 59.2)	0.79 (0.48 to 1.30)	0.90 (0.53 to 1.52)
35–44 years	36.2 (32.2 to 40.2)	62.9 (56.2 to 69.6)	1.27 (0.79 to 2.06)	1.47 (0.88 to 2.44)
45–54 years	38.2 (34.1 to 42.3)	62.5 (55.8 to 69.1)	1.25 (0.78 to 2.02)	1.26 (0.76 to 2.07)
≥55 years	33.6 (28.1 to 39.0)	57.1 (47.3 to 66.9)	Reference	Reference
Gender				
Female	22.6 (19.5 to 25.8)	64.5 (56.7 to 72.3)	Reference	Reference
Male	30.4 (28.3 to 32.5)	56.7 (52.5 to 60.9)	0.72 (0.50 to 1.03)	0.72 (0.49 to 1.07)
Type of homelessness				
Housed—own tenancy*	23.6 (18.1 to 29.2)	61.6 (48.4 to 74.8)	Reference	Reference
Hostel or supported accommodation	28.5 (26.2 to 30.8)	67.5 (63.0 to 72.1)	1.30 (0.73 to 2.30)	1.38 (0.76 to 2.47)
Bed & breakfast or other temporary accommodation	22.8 (15.4 to 30.1)	46.1 (27.5 to 64.8)	0.53 (0.22 to 1.31)	0.52 (0.21 to 1.30)
Emergency accommodation	27.5 (21.1 to 34.0)	49.3 (35.3 to 63.3)	0.61 (0.28 to 1.30)	0.64 (0.30 to 1.40)
Rough sleeping	34.3 (28.2 to 40.3)	28.3 (17.9 to 38.7)	0.25 (0.12 to 0.50)	0.26 (0.12 to 0.54)
Sofa surfing	28.6 (21.9 to 35.3)	43.8 (29.9 to 57.7)	0.49 (0.23 to 1.04)	0.53 (0.24 to 1.17)
Other	28.3 (18.3 to 38.3)	59.0 (38.4 to 79.6)	0.90 (0.33 to 2.41)	0.89 (0.33 to 2.43)
Number of types of homelessness experienced†	‒	‒	0.85 (0.73 to 0.99)	0.86 (0.74 to 1.00)

With respective other variables used for adjustment. For number of types of homelessness experienced, adjusted for age and gender.

* People supported by homelessness support services having very recently moved into a property.

† Options included: stayed at a hostel, foyer, refuge, night shelter or bed & breakfast hotel or any other type of homelessness service; sofa surfed; slept rough; applied to the council as homeless; none of the above.

OR_adj_, adjusted OR.

Of those self-identifying as having an alcohol problem, approximately half stated that they currently had a problem (54.9% (95% CI 51.1% to 58.7%)) and the other half that they were in recovery (45.1% (95% CI 41.3% to 48.9%); online supplemental table S5). Among those in recovery, 48.3% (95% CI 42.5% to 54.0%) stated that they received support appropriate to their needs, while among those with a current problem, this figure was only 29.9% (95% CI 25.1% to 34.6%).

### Type of support

Among those self-identifying as having an alcohol problem and receiving support, nearly half stated attending mutual aid groups or receiving advice and information (table 4). About one-third reported receiving counselling/psychological support or help from others who had been through a similar experience. Around 1 in 10 said they had undergone inpatient detoxification or residential rehabilitation.

**Table 4:** Type of support received by those with a self-identified alcohol problem who receive support for it (n=524; options not mutually exclusive)

Type of support	% (95% CI)
Advice and information	44.6 (39.3 to 49.9)
Mutual aid	46.1 (41.2 to 51.0)
Community prescribing	15.5 (11.5 to 19.6)
Counselling or psychological support	30.6 (25.8 to 35.4)
Day programme in community	21.7 (17.3 to 26.1)
Detoxification	13.1 (9.8 to 16.3)
Residential rehabilitation	12.3 (8.8 to 15.9)
Aftercare	12.0 (8.5 to 15.4)
Peer support	30.7 (26.1 to 35.4)
Other support	17.0 (12.8 to 21.1)

Individuals aged 18–34 years had significantly higher odds of receiving psychological or community-based interventions than those aged 35 and over (OR_adj_=1.53 (95% CI 1.04 to 2.27); table 5). Furthermore, each additional type of homelessness experienced was associated with increased odds of receiving such interventions (OR_adj_=1.20 (95% CI 1.01 to 1.43)). No other subgroup differences reached statistical significance, although there was some indication that males may have lower odds of receiving psychological/community-based interventions than females (OR_adj_=0.73 (95% CI 0.49 to 1.09)) and people experiencing unsheltered or hidden homelessness may have lower odds of receiving information or peer-led support than those in their own tenancy (OR_adj_=0.41 (95% CI 0.14 to 1.17)).

**Table 5:** ORs for receiving different types of support by age, gender and type of homelessness (n=524)

Characteristics		Information and peer-led support	Psychological and community-based interventions	Medical and residential treatment
Age (ref. ≥35)	OR	1.02 (0.65 to 1.60)	1.55 (1.06 to 2.28)	1.25 (0.83 to 1.88)
	OR_adj_	1.07 (0.67 to 1.69)	1.53 (1.04 to 2.27)	1.33 (0.88 to 2.02)
Gender (ref. female)	OR	0.92 (0.58 to 1.47)	0.70 (0.47 to 1.04)	0.99 (0.64 to 1.52)
	OR_adj_	0.95 (0.59 to 1.53)	0.73 (0.49 to 1.09)	1.06 (0.68 to 1.66)
Temporary or supported housing (ref. own tenancy)	OR	0.63 (0.26 to 1.55)	1.03 (0.54 to 1.98)	0.68 (0.35 to 1.33)
	OR_adj_	0.63 (0.25 to 1.56)	1.02 (0.53 to 1.98)	0.65 (0.33 to 1.27)
Unsheltered or hidden homelessness (ref. own tenancy)	OR	0.42 (0.15 to 1.17)	0.94 (0.42 to 2.11)	0.83 (0.36 to 1.92)
	OR_adj_	0.41 (0.14 to 1.17)	0.89 (0.39 to 2.04)	0.76 (0.33 to 1.79)
Other type (ref. own tenancy)	OR	0.68 (0.16 to 2.88)	1.11 (0.36 to 3.46)	1.68 (0.53 to 5.29)
	OR_adj_	0.68 (0.16 to 2.89)	1.16 (0.37 to 3.65)	1.74 (0.55 to 5.55)
Number of types of homelessness experienced*	OR	0.95 (0.78 to 1.15)	1.19 (1.01 to 1.41)	1.04 (0.87 to 1.24)
	OR_adj_	0.95 (0.78 to 1.16)	1.20 (1.01 to 1.43)	1.01 (0.84 to 1.21)
In recovery (ref. current alcohol problem)	OR	1.77 (1.12 to 2.81)	1.13 (0.77 to 1.68)	0.88 (0.57 to 1.37)
	OR_adj_	1.59 (0.97, 2.61)	1.14 (0.75 to 1.74)	0.92 (0.58 to 1.47)
Support that meets needs (ref. support but would like more)	OR	1.52 (0.96 to 2.41)	1.02 (0.68 to 1.53)	0.87 (0.55 to 1.36)
	OR_adj_	1.37 (0.83 to 2.24)	0.95 (0.61 to 1.47)	0.92 (0.58 to 1.47)

For number of types of homelessness experienced, adjusted for age and gender. For ‘In recovery’ and ‘Support that meets needs’, adjusted for age, gender, type of homelessness and respective other of the two.

* Options included: stayed at a hostel, foyer, refuge, night shelter or bed & breakfast hotel or any other type of homelessness service; sofa surfed; slept rough; applied to the council as homeless; none of the above.

OR_adj_, adjusted OR.

### Population estimates

When weighted according to different types of homelessness, prevalence of alcohol use was 78.6% compared with 83.4% (95% CI 82.4% to 84.4) in private households (HSE 2022); drinking above guideline recommendations was 35.2% compared with 23.9% (95% CI 22.7% to 25.1%); and possible AUD was 21.9% compared with 4.8% (95% CI 4.2% to 5.5%). Despite increased prevalence, since the absolute numbers of people experiencing homelessness is relatively small, accounting for those experiencing homelessness did not notably impact overall prevalence estimates at the population level: 83.4%, 24.0% and 4.9% (see online supplemental file 1 for details).

### Unplanned and sensitivity analyses

Mean weekly alcohol consumption (excluding those abstaining) was highest among people sleeping rough (median: 43.4 units, IQR 5.2–141.3; see online supplemental table S6). Weighted across homelessness type, consumption was higher (9.7, IQR 1.7–46.6) than in private households (6.0, IQR 1.4–16.4). A pronounced difference in consumption was also observed among those with possible AUD (homelessness: 97.5, IQR 65.0–149.0 vs private households: 63.8, IQR 50.7–83.4).

The results of the complete case analysis were comparable to those of the primary analysis with multiple imputations, except that the 95% CIs were wider due to the smaller sample size (online supplemental tables S7-S12).

In the analysis using 2018 HSE and Homelessness Monitor data, the prevalence of alcohol use weighted by type of homelessness was 78.6% compared with 82.6% (95% CI 81.6% to 83.5%) in private households; drinking above guideline recommendations was 35.5% compared with 22.5% (95% CI 21.5% to 23.5%); and possible AUD was 22.2% compared with 4.1% (95% CI 3.6% to 4.5%).

## Discussion

### Summary

Based on HHNA data, 79% of people experiencing homelessness consumed alcohol, 31% exceeded guideline recommendations and 18% met criteria for possible AUD. After weighting by type of homelessness, these estimates were 78%, 35% and 22%, respectively. The AUD figure broadly aligns with international data^7^ and is about four times higher than the rates in the general population. While the proportion consuming any alcohol was similar to those living in private households, exceeding guidelines and AUD were substantially more common among people experiencing homelessness. This had little impact on overall population-level estimates due to the relatively small size of this group. However, 22% equates to approximately 50 000 people who experienced homelessness in England with possible AUD in 2022—a number possibly to have risen given social and affordable housing shortages, inflation and welfare cuts.^1^

Among those experiencing homelessness, individuals sleeping rough had a higher prevalence of exceeding guidelines and possible AUD. Odds increased with the number of homelessness types experienced. Young adults (18–24) had lower prevalence than older groups, possibly reflecting increased drinking over time the longer someone experiences housing instability or heightened risk of chronic homelessness among those with AUD.^24^

One-third self-identified as ever having an alcohol problem; roughly half reported a current problem and half were in recovery. Just over half had received support. Of those in recovery, about half felt support met their needs versus one-third with a current problem, likely reflecting self-selection into the recovery group by those who have found treatment helpful. Young adults had approximately 50% higher odds of receiving psychological or community-based interventions than those aged ≥35. The reason for this difference is unclear. Younger people may be more likely to engage with such services, or the services are more tailored towards them. In contrast, individuals with a longer history of alcohol dependence may have stopped using them after finding they did not effectively support their long-term recovery.

Notably, people sleeping rough who identified an alcohol problem had approximately four times lower odds of receiving support than those in their own tenancy. People who sleep rough represent some of the most socially excluded individuals. They also may face additional competing priorities and survival needs, as well as practical barriers to treatment engagement (eg, transportation or storing belongings). Therefore, it is plausible this difference could be indicative of the level of contact individuals have with different services. It may also indicate that when people sleep outside their substance use is deprioritised—at least temporarily—until more secure and stable accommodation has been found. Indeed, the most reported types of support were advice or information and mutual aid groups such as Alcoholics Anonymous. Support from the latter lies outside the formal national healthcare system.

### Policy implications

The results of this study highlight the needs gap: While the prevalence of possible AUD is relatively high, a substantial proportion of participants expressed the desire for (more adequate) support. The results also suggest that those experiencing the most unstable housing arrangements (having experienced various types of homelessness and/or currently sleeping rough) are the most at risk of AUD and of not receiving adequate support. The UK Government has set a mandate and treatment plan, ‘All Our Health’, which advises that all health professionals regardless of the primary issue of presentation offer holistic support to improve health outcomes among socially excluded groups.^25^ However, addressing AUD in the context of homelessness can be complex. Further, some people who are experiencing homelessness may not use mainstream healthcare often, creating additional barriers to access.

To this end, the UK Government has increased funding for drug and alcohol treatment and recovery in 2025–2026. This includes £310 million in additional targeted grants.^26^ A further £58.7 million has been allocated for specific programmes to fund local drug and alcohol treatment and support for people sleeping rough or at risk of sleeping rough.^27^ These funding increases represent an important step towards addressing the needs gap. Previously, substance use services were only funded through public health grants paid to local authorities from the Department for Health and Social Care, but there was no ring-fenced funding for these specific services. It is estimated that drug and alcohol services for adults have been cut in real terms by 25% between 2015/2016 and 2025/2026.^28^ However, it remains to be seen whether these additional funds will be sufficient against a backdrop of continual social and economic conditions in England, especially for the most socially excluded. Targeted, accessible, and adequately resourced interventions will be critical to ensuring that those experiencing the greatest disadvantage are effectively reached.

### Strengths and limitations

A key strength of this study is the use of a relatively large dataset drawn from a population that is often overlooked, with broad representation across different forms of homelessness. Although the dataset contained a substantial proportion of missing values, we addressed this by applying multiple imputations to reduce the risk of bias. To test the robustness of this approach, we also conducted a complete case analysis.

Important limitations of the study are that the sample was not fully representative of all people experiencing homelessness and there is a risk of selection bias: For example, about 50% of those taking part in the HHNA did not consent to their data being used for research purposes and were therefore not included in this study, the survey used convenience sampling and data were collected over a 6-year period. Additionally, some subgroup analyses were based on relatively small sample sizes, resulting in greater uncertainty around the corresponding estimates and we were unable to include gender minorities in the analysis. Given the cross-sectional nature of the data, causality cannot be inferred.

For the reassessment of national prevalence figures, several assumptions were necessary, which may have led to inaccurate estimates. For example, we used external research^18^ to estimate the number of people who experienced different types of homelessness in 2022, and we were unable to directly map the homelessness categories included in the HHNA survey to those used in external sources, limiting comparability. Furthermore, as this study highlights, many individuals experience complex and overlapping patterns of homelessness, rather than a single type. Another challenge was the lack of precise figures on the number of adults living in private households in England, which further constrained our ability to produce accurate population-level estimates.

## Conclusion

While there are no notable differences in any drinking, people experiencing homelessness have higher rates of exceeding guidelines and particularly possible AUD than people in private households. Individuals sleeping rough are particularly vulnerable—not only to experiencing AUD but also to being underserved by existing treatment systems. While the UK Government has recently increased funding for drug and alcohol services, including dedicated funds for people who are sleeping rough, it remains to be seen whether these measures are sufficient to address the scale and complexity of support needs among this population.

## Supplementary material

10.1136/bmjph-2026-004924 online supplemental file 1

## Data Availability

Data may be obtained from a third party and are not publicly available.
