# Supplementary material for: Alcohol use and support receipt among people experiencing homelessness in England: a cross-sectional analysis with comparison to private household populations
Source: BMJ Public Health. 2026 Aug 24;4(3):e004924. doi: 10.1136/bmjph-2026-004924 (PMC13504531; doi:10.1136/bmjph-2026-004924)
Supplement: Supplementary file 1 [file bmjph-4-3-s001.pdf]

Supplementary material

Article: Alcohol use among people experiencing homelessness and reassessment of population prevalence of any and harmful alcohol consumption in England

Table of Contents

1. **Measures for homelessness** ... 1
2. **Missing data and multiple imputations** ... 3
3. **Additional analyses** ... 5
4. **Calculations for population estimates** ... 6
5. **Complete case analysis** ... 7
6. **References** ... 11

1. Measures for homelessness

Type of homelessness

Question from Homeless Health Needs Audit (HHNA) survey: "Where are you currently sleeping? (if this frequently changes, please say where you slept last night)"

Answer options:

a) Housed – in own tenancy
b) In a hostel or supported accommodation
c) In B&B or other temporary accommodation
d) In emergency accommodation, e.g., night shelter, refuge
e) Rough sleeping
f) Sleeping on somebody's sofa/floor
g) Squatting
h) Staying with friends/family
i) Vehicle or caravan on the side of the road or car park
j) Other
k) No answer

Answer option k was considered missing. During the COVID-19 pandemic, there were some additional answer options that were selected by few people ("In NASS accommodation" [n=1], "In temporary Hotel accommodation (due to Everyone In)" [n=2]). We included these in the 'other category'. Answer options f (n=175) and h (n=14) were combined to 'sofa surfing'. Answer option g (n=14) was included in the 'other' category. Answer option i (n=10) was included 'rough sleeping' because the UK Government includes people sleeping in cars in their definition of rough sleeping,[1] and people sleeping in cars typically have similar experiences with accessing services as people experiencing other forms of rough sleeping such as sleeping in a tent or in the open air.

In the protocol, we stated that for RQ4 we would include those who answered option i in a category ('unconventional accommodation') together with option g. However, as the numbers were small and we were otherwise unable to perform multiple imputations as specified, we deviated from the protocol.

This resulted in the following groups for RQs 1 and 2: housed – in own tenancy; in a hostel or supported accommodation; in B&B or other temporary accommodation; in emergency accommodation; rough sleeping; sofa surfing; other type.

For RQ3, we used these groups: housed – own tenancy; temporary or supported housing (combining: 'in a hostel or supported accommodation'; 'in B&B or other temporary accommodation'; 'in emergency

accommodation'); unsheltered or hidden homelessness (combining: 'rough sleeping', 'sofa surfing'); other type.

For RQ4, we used these groups: rough sleeping; hostels; unsuited temporary accommodation; sofa surfing; unconventional accommodation.

Estimates for number of people experiencing different forms of core homelessness in England in 2022[2,3]

- Rough sleeping (defined as "sleeping in the open, e.g., in streets, parks, carparks, doorways"): n=11,719
- Unconventional accommodation (defined as "sleeping in places/spaces not intended as normal residential accommodation, e.g., cars, vans, lorries, caravans/motor homes, tents, boats, sheds, garages, industrial/commercial premises"): n=17,539
- Hostels, etc. (defined as "communal emergency and temporary accommodation primarily targeted at homeless people including hostels, refuges and shelters"): n=46,733
- Unsuited temporary accommodation (defined as "homeless households placed in temporary accommodation of certain types, B&B, private non-self-contained licensed/nightly let, and out of area placements"): n=26,923
- Sofa surfing (defined as "individuals or family groups staying temporarily (expecting or wanting to move) with another household, excluding nondependent children of hist households and students, who are also overcrowded on the bedroom standard") n=139,278

That results in a total of n=242,191.

We mapped these to the answer options of the question on type of homelessness (see above) as follows:

- Rough sleeping – answer options e, i
- Unconventional accommodation – answer options g, j
- Hostels, etc. – answer options b, c
- Unsuited temporary accommodation – answer option d
- Sofa surfing – answer options f and h

The groups might not map ideally onto each other, but considering small numbers for some groups in the survey and relatively small variation in prevalence estimates between some groups, we deemed this approach acceptable.

History of homelessness

Question from the HHNA survey: "Have you ever (in your lifetime) done any of the following?"

Answer options (not mutually exclusive):

a) Stayed at a hostel, foyer, refuge, night shelter or B&B hotel, or any other type of homelessness service
b) Stayed with friends or relatives because had no home of own ('sofa surfed')
c) Slept rough
d) Applied to the council as homeless
e) None of the above
f) No answer

Answer option f was considered missing. We created four binary variables, each indicating whether the person ever experienced the respective form of homelessness: 'Stayed at a hostel, foyer, refuge, night shelter or B&B hotel, or any other type of homelessness service', 'Stayed with friends or relatives because had no home of own ('sofa surfed'), 'Slept rough', 'Applied to the council as homeless'.

If people reported currently staying in a hostel, sofa surfing, or sleeping rough, respectively, we classified them accordingly as having ever experienced the respective form of homelessness for this question.

2. Missing data and multiple imputations

We removed 14 participants from the dataset due to their gender being reported in another way, resulting in a sample of 2652 participants.

Table S1: Missing values for each variable included in the analysis (N=2,652).

	Missing values, n (%)
Age	0 (0)
Gender	20 (0.8)
Type of homelessness	20 (0.8)
History of homelessness – hostel, etc	69 (2.6)
History of homelessness – sofa surfing	86 (3.2)
History of homelessness – rough sleeping	98 (3.7)
History of homelessness – applied to council	76 (2.9)
How often alcohol in past year	260 (9.8)
How many units on typical day[1]	133 (7.0)
Alcohol problem	233 (8.8)
Receiving support[2]	32 (4.6)
Type of support received – information, etc[3]	28 (7.2)
Type of support received – mutual aid[3]	25 (6.4)
Type of support received – community prescription[3]	31 (7.9)
Type of support received – counselling, etc[3]	29 (7.4)
Type of support received – day programme[3]	29 (7.4)
Type of support received – detox[3]	32 (8.2)
Type of support received – residential rehabilitation[3]	30 (7.7)
Type of support received – aftercare[3]	32 (8.2)
Type of support received – peer support[3]	28 (7.2)
Type of support received – other support[3]	0 (0)

[1] Only asked to those who reported drinking in past year (n=1892). [2] Only asked to those who reported alcohol problem (n=694). [3] Only asked to those who reported alcohol problem and receiving support (n=391).

We used the mice package in R[4,5], setting the function to 20 sets of imputations and 5 iterations.

Table S2: Variables with missing values and how they were imputed (in weighted analysis, survey weight was additionally added to all imputation models).

Variable with missing values	Imputation method and conditions for imputation	Variables used for imputation
Gender	Logistic regression	Age, type of homelessness, all variables related to history of homelessness, how often alcohol in past year, how many units on a typical day, alcohol problem
Type of homelessness	Polytomous logistic regression	Age, gender, all variables related to history of homelessness, how often alcohol in past year, how many units on a typical day, alcohol problem
History of homelessness (each variable separate)	Logistic regression	Age, gender, type of homelessness, all other variables related to history of homelessness, how often alcohol in past year, how many units on a typical day, alcohol problem
How often alcohol in past year	Polytomous logistic regression	Age, gender, type of homelessness, all variables related to history of homelessness, how many units on a typical day, alcohol problem
How many units on typical day[1]	Predictive mean matching; do not impute if not drinking in past year	Age, gender, type of homelessness, all other variables related to history of homelessness, how often alcohol in past year, alcohol problem
Alcohol problem	Polytomous logistic regression	Age, gender, type of homelessness, all other variables related to history of homelessness, how often alcohol in past year, how many units on a typical day, alcohol problem
Receiving support[2]	Polytomous logistic regression; do not impute if no alcohol problem	Age, gender, type of homelessness, all variables related to history of homelessness, how often alcohol in past year, how many units on a typical day
Type of support received (each variable separate)	Logistic regression; do not impute of no alcohol problem or not received support	Age, gender, type of homelessness, all variables related to history of homelessness, how often alcohol in past year, how many units on a typical day

[1] Only asked to those who reported drinking in past year. [2] Only asked to those who reported alcohol problem. [3] Only asked to those who reported alcohol problem and receiving support.

3. Additional analyses

Table S3: Prevalence estimates of alcohol use, exceeding guideline recommendations, and possible alcohol use disorder in subgroups, adjusted for the respective other characteristics (age, gender, type of homelessness).

Characteristic	**Adjusted prevalence, % (95% CI)**		
	Alcohol use	Exceeding guideline recommendations	Possible alcohol use disorder
18-24 years	79.0 (74.8, 82.8)	18.5 (15.1, 22.5)	8.5 (6.2, 11.6)
25-34 years	78.1 (73.8, 81.8)	29.6 (25.4, 34.1)	17.5 (14.2, 21.5)
35-44 years	77.3 (72.9, 81.2)	32.9 (28.3, 37.9)	23.9 (19.9, 28.5)
45-54 years	74.9 (69.8, 79.5)	33.6 (28.9, 38.6)	23.1 (18.8, 28.0)
≥55 years	65.6 (58.9, 71.8)	31.6 (25.8, 38.0)	22.8 (17.6, 29.1)
Female	73.5 (69.2, 77.4)	25.3 (21.6, 29.4)	17.3 (14.0, 21.2)
Male	77.0 (73.9, 79.8)	32.7 (29.5, 36.2)	19.2 (16.4, 22.3)
Housed – in own tenancy	73.2 (66.9, 78.7)	22.6 (17.7, 28.5)	12.2 (8.6, 17.0)
In a hostel or supported accommodation	78.1 (75.4, 80.6)	28.2 (25.5, 31.0)	16.2 (14.1, 18.6)
In B&B or other temporary accommodation	70.9 (62.0, 78.5)	22.2 (15.6, 30.6)	14.8 (9.4, 22.4)
In emergency accommodation	76.2 (68.4, 82.5)	26.7 (20.3, 34.3)	17.4 (12.2, 24.1)
Rough sleeping	79.7 (73.4, 84.8)	43.5 (36.6, 50.7)	35.5 (29.0, 42.7)
Sofa surfing	77.5 (70.1, 83.4)	37.0 (30.0, 44.6)	23.4 (17.5, 30.6)
Other type	70.2 (58.2, 79.9)	25.1 (16.6, 36.0)	14.3 (8.3, 23.7)

Reference groups used for adjustment: age ≥55 years, female, housed – in own tenancy

Table S4: Distribution of type of homelessness by gender.

**Type of homelessness**	**Females, % (95% CI)**	**Males, % (95% CI)**
Housed – in own tenancy	14.4 (11.9, 17.0)	7.0 (5.9, 8.2)
In a hostel or supported accommodation	54.5 (50.8, 58.1)	60.2 (58.0, 62.4)
In B&B or other temporary accommodation	6.2 (4.4, 7.9)	4.7 (3.7, 5.6)
In emergency accommodation	8.7 (6.6, 10.7)	7.0 (5.9, 8.2)
Rough sleeping	4.8 (3.2, 6.4)	11.1 (9.7, 12.5)
Sofa surfing	7.6 (5.6, 9.5)	7.1 (5.9, 8.2)
Other type	3.9 (2.5, 5.3)	2.9 (2.1, 3.6)

Table S5: Percentage of people with self-identified alcohol problem – current or in recovery (n=710) and whether they receive adequate support.

	**% (95% CI)**		
	All who self-identified alcohol problem	Current alcohol problem	In recovery
All who self-identified alcohol problem	–	54.9 (51.1, 58.7)	45.1 (41.3, 48.9)
No support needed	26.0 (22.7, 29.4)	19.2 (15.1, 23.2)	34.4 (29.0, 39.9)
No support but would be helpful	15.9 (13.0, 18.8)	23.8 (19.3, 28.2)	6.3 (3.2, 9.3)
Support that meets needs	38.2 (34.4, 42.0)	29.9 (25.1, 34.6)	48.3 (42.5, 54.0)
Support but would like more	19.9 (16.9, 22.9)	27.2 (22.7, 31.7)	11.0 (7.4, 14.7)

4. Calculations for population estimates

We estimated the number of people experiencing homelessness in England in 2022 (time the Health Survey for England was conducted) and adjust the Health Survey for England prevalence estimates by accounting for the number of people using alcohol, exceeding guidelines, and having possible alcohol use disorder among those experiencing homelessness to get the population prevalence $Prevalence_{\mathrm{p}}$using the following formula:

$$Prevalence_p = \frac{n_r * prevalence_r + n_h * prevalence_h}{n_r + n_h}$$

Where n_r is the residential adult population (living in private households), n_h is the number of adults experiencing homelessness, $prevalence_r$ is the percentage estimate of alcohol use, exceeding guidelines, or possible alcohol use disorder, respectively, among the usual resident adult population (based on Health Survey for England data), and $prevalence_h$ the percentage estimate of alcohol use, exceeding guidelines, or possible alcohol use disorder, respectively, among adults experiencing homelessness (based on HHNA data).

Using 2022 data (Health Survey for England and Homelessness Monitor)

- Number of people experiencing homelessness in England in 2022: n_h **= 242,192** (see *1. Measures for homelessness*)
- Number of people aged 18 and over living in private households in England in 2022:
  - estimated 23,626,000 households in England in 2022, with 2.38 residents per household[6]
  - England population-mid 2022[7]: 57,106,398
  - estimated 45,219,492 people aged 18 and over in England in 2022[7]
  - proportion of all living in private households multiplied by all adults:
    n_r = (23,626,000*2.38) / 57,106,398 * 45,219,492 = **44,525,424**

Alcohol use

- weighted prevalence of drinking alcohol among people experiencing homelessness:
  $prevalence_h$ = (11719*0.8158076 + 17539*0.6992919 + 46733*0.7892063 + 26923*0.7794927 +139278*0.7949772) / 242,192 = **0.78622**
- prevalence of drinking alcohol among people living in private households:
  $prevalence_r$ = **0.83403**
- adjusted prevalence of drinking alcohol for total population: $Prevalence_p$= **0.83377**

Exceeding guideline recommendations

- weighted prevalence of exceeding guideline recommendations among people experiencing homelessness: $prevalence_h$ = (11719*0.4744560 + 17539*0.2817520 + 46733*0.2966806 + 26923*0.2847895 + 139278*0.3823004) / 242,192 = **0.35212**
- prevalence of exceeding guideline recommendations among people living in private households:
  $prevalence_r$= **0.23922**
- adjusted prevalence of drinking alcohol for total population: $Prevalence_p$ = **0.23983**

Possible alcohol use disorder

- weighted prevalence of possible alcohol use disorder among people experiencing homelessness:
  $prevalence_h$ = (11719*0.3782404 + 17539*0.1685794 + 46733*0.1700626 + 26923*0.1824770 + 139278*0.2346147) / 242,192 = **0.21853**
- prevalence of possible alcohol use disorder among people living in private households:
  $prevalence_r$= **0.048367**
- adjusted prevalence of drinking alcohol for total population: $Prevalence_p$ = **0. 04929**

Using 2018 data (Health Survey for England and Homelessness Monitor)

Estimates for number of people experiencing different forms of core homelessness in England in 2018[8]

- Rough sleeping: n=13,729
- Unconventional accommodation: n=17,556
- Hostels, etc.: n=36,415
- Unsuited temporary accommodation: n=18,517
- Sofa surfing: n=114,392
- Total number of people experiencing homelessness in England in 2018: n_h **= 200,609**

Number of people aged 18 and over living in private households in England in 2018[9,10]:

- estimated 23,204,000 households in England in 2018, with 2.38 residents per household[10]
- England population-mid 2018[9]: 55,977,178
- estimated 44,022,560 people aged 18 and over in England in 2018[9]
- proportion of all living in private households multiplied by all adults: n_r = (23,204,000*2.38) / 55,977,178 * 44,022,560 = **43,431,428**

Alcohol use

- weighted prevalence of drinking alcohol among people experiencing homelessness: $prevalence_h$ = (13729*0.8158076 + 17556*0.6992919 + 36415*0.7892063 + 18517*0.7794927 + 114392*0.7949772) / 200,609 = **0.78555**
- prevalence of drinking alcohol among people living in private households: $prevalence_r$ = **0.82559**
- adjusted prevalence of drinking alcohol for total population: $Prevalence_p$= **0.82541**

Exceeding guideline recommendations

- weighted prevalence of exceeding guideline recommendations among people experiencing homelessness: $prevalence_h$ = (13729*0.4744560 + 17556*0.2817520 + 36415*0.2966806 + 18517*0.2847895 + 114392*0.3823004) / 200,609 = **0.35527**
- prevalence of exceeding guideline recommendations among people living in private households: $prevalence_r$= **0.22483**
- adjusted prevalence of drinking alcohol for total population: $Prevalence_p$ = **0.22543**

Possible alcohol use disorder

- weighted prevalence of possible alcohol use disorder among people experiencing homelessness: $prevalence_h$ = (13729*0.3782404 + 17556*0.1685794 + 36415*0.1700626 + 18517*0.1824770 + 114392*0.2346147) / 200,609 = **0.22213**
- prevalence of possible alcohol use disorder among people living in private households: $prevalence_r$= **0.040787**
- adjusted prevalence of drinking alcohol for total population: $Prevalence_p$ = **0. 04162**

Table S6: Mean weekly alcohol consumption among people experiencing homelessness and those in private households, excluding those who abstain from alcohol. Values were capped at 700 units per week in line with other research.[11]

**Housing type**	**Geometrical Mean (95% CI)**	**Median (IQR)**
In emergency accommodation	6.3 (5.3, 7.4)	7.9 (0.9, 48.1)
Rough sleeping	23.7 (20.8, 27.0)	43.4 (5.2, 141.3)
Sofa surfing	13.4 (11.9, 15.1)	21.1 (3.0, 64.7)
Hostel	7.6 (7.0, 8.2)	9.2 (1.5, 41.6)
Other	8.5 (6.6, 11.0)	8.9 (2.0, 60.5)
All experiencing homelessness, weighted by type	8.0 (7.5, 8.6)	9.7 (1.7, 46.6)
Private households (HSE 2022)	4.1 (3.8, 4.3)	6.0 (1.4, 16.4)
**Among those with possible alcohol use disorder**		
All experiencing homelessness, weighted by type	105.0 (103.0, 108.0)	97.5 (65.0, 149.0)
Private households (HSE 2022)	68.4 (64.5, 72.5)	63.8 (50.7, 83.4)

5. Complete case analysis

Table S7: Sociodemographic characteristics of participants (N=1,993).

Characteristic	Category	**% (95% CI)**
Age	18-24 years	20.8 (19.0, 22.6)
	25-34 years	23.2 (21.4, 25.1)
	35-44 years	22.1 (20.3, 23.9)
	45-54 years	21.8 (20.0, 23.6)
	≥55 years	12.1 (10.7, 13.5)
Gender	Female	26.0 (24.1, 28.0)
	Male	74.0 (72.0, 75.9)
Type of homelessness	Housed – in own tenancy	9.1 (7.9, 10.4)
	In a hostel or supported accommodation	59.7 (57.5, 61.8)
	In B&B or other temporary accommodation	4.6 (3.7, 5.5)
	In emergency accommodation	7.1 (6.0, 8.3)
	Rough sleeping	8.8 (7.5, 10.0)
	Sofa surfing	7.6 (6.5, 8.8)
	Other type	3.1 (2.3, 3.8)
Number of different types of homelessness experienced[1]	0	2.7 (2.0, 3.4)
	1	12.4 (10.9, 13.8)
	2	19.2 (17.4, 20.9)
	3	28.8 (26.8, 30.8)
	4	37.0 (34.9, 39.1)

[1]Options included: stayed at a hostel, foyer, refuge, night shelter or B&B hotel, or any other type of homelessness service; sofa surfed; slept rough; applied to the council as homeless; none of the above.

Table S8: Raw prevalence of alcohol use, exceeding guideline recommendations, and possible alcohol overall (N=1,993) and among subgroups.

Characteristic	Raw prevalence, % (95% CI)		
	Alcohol use	Exceeding guideline recommendations	Possible alcohol use disorder
Overall	78.4 (76.6, 80.2)	32.1 (30.0, 34.1)	19.6 (17.9, 21.4)
18-24 years	79.5 (75.6, 83.4)	20.0 (16.2, 23.9)	9.2 (6.4, 12.0)
25-34 years	81.4 (77.9, 85.0)	32.8 (28.6, 37.1)	18.8 (15.2, 22.3)
35-44 years	78.9 (75.0, 82.7)	35.9 (31.4, 40.4)	25.5 (21.4, 29.5)
45-54 years	78.9 (75.0, 82.7)	38.2 (33.6, 42.7)	23.7 (19.7, 27.7)
≥55 years	68.9 (63.0, 74.7)	33.2 (27.2, 39.1)	22.1 (16.0, 26.3)
Female	75.9 (72.2, 79.6)	24.5 (20.8, 28.2)	16.8 (13.5, 20.0)
Male	79.2 (77.2, 81.3)	34.7 (32.3, 37.2)	20.6 (18.6, 22.7)
Housed – in own tenancy	73.6 (67.2, 80.0)	24.2 (18.0, 30.4)	13.2 (8.3, 18.1)
In a hostel or supported accommodation	79.1 (76.8, 81.5)	30.9 (28.2, 33.5)	17.4 (15.3, 19.6)
In B&B or other temporary accommodation	70.7 (61.3, 80.0)	26.1 (17.1, 35.1)	19.6 (11.5, 27.7)
In emergency accommodation	80.3 (73.7, 86.8)	28.9 (21.4, 36.3)	19.0 (12.6, 25.5)
Rough sleeping	80.6 (74.7, 86.4)	46.9 (39.5, 54.3)	37.7 (30.5, 44.9)
Sofa surfing	82.2 (76.2, 88.3)	40.8 (33.0, 48.6)	25.0 (18.1, 31.9)
Other type	68.9 (57.2, 80.5)	31.1 (19.5, 42.8)	18.0 (8.4, 27.7)

Table S9: Percentage of people self-identifying ever having had an alcohol problem (N=1,993), and among these (n=538), percentage who have received support for it overall and among subgroups, as well as unadjusted and adjusted odds ratios for receiving support.

Characteristic	Self-identified alcohol problem	Support		
	**% (95% CI)**	**% (95% CI)**	**OR (95% CI)**	**OR_{adj}[1] (95% CI)**
Overall	27.0 (25.0, 28.9)	56.9 (52.7, 61.1)	–	–
18-24 years	6.8 (4.3, 9.2)	46.4 (28.0, 64.9)	0.80 (0.33, 1.92)	0.84 (0.33, 2.13)
25-34 years	26.1 (22.1, 30.1)	49.6 (40.7, 58.5)	0.91 (0.51, 1.62)	1.04 (0.56, 1.92)
35-44 years	34.3 (29.9, 38.8)	59.6 (51.8, 67.4)	1.36 (0.78, 2.39)	1.59 (0.88, 2.87)
45-54 years	37.5 (32.9, 42.0)	63.8 (56.4, 71.2)	1.63 (0.93, 2.85)	1.69 (0.95, 3.03)
≥55 years	31.1 (25.3, 37.0)	52.0 (40.7, 63.3)	Reference	Reference
Female	22.0 (18.4, 25.5)	64.9 (56.2, 73.7)	Reference	Reference
Male	28.8 (26.5, 31.1)	54.7 (50.0, 59.5)	0.65 (0.42, 1.00)	0.68 (0.43, 1.07)
Housed – in own tenancy	23.1 (17.0, 29.2)	61.9 (47.2, 76.6)	Reference	Reference
In a hostel or supported accommodation	27.3 (24.8, 29.9)	64.9 (59.7, 70.1)	1.14 (0.57, 2.20)	1.16 (0.57, 2.29)
In B&B or other temporary accommodation	23.9 (15.2, 32.6)	45.5 (24.6, 66.3)	0.51 (0.18, 1.46)	0.48 (0.16, 1.40)
In emergency accommodation	23.2 (16.3, 30.2)	48.5 (31.4, 65.5)	0.58 (0.23, 1.46)	0.60 (0.23, 1.56)
Rough sleeping	33.1 (26.2, 40.1)	29.3 (17.6, 41.0)	0.26 (0.11, 0.59)	0.27 (0.11, 0.63)
Sofa surfing	28.3 (21.1, 35.4)	41.9 (27.1, 56.6)	0.44 (0.18, 1.05)	0.47 (0.19, 1.14)
Other type	24.6 (13.8, 35.4)	53.3 (28.1, 78.6)	0.70 (0.21, 2.38)	0.63 (0.18, 2.19)
Number of different types of homelessness experienced[2]	–	–	0.84 (0.70, 1.00)	0.84 (0.70, 1.00)

[1]adjusted for the respective other co-variates – age, gender, type of homelessness. For number of types of homelessness experienced, adjusted for age and gender. [2]Options included: stayed at a hostel, foyer, refuge, night shelter or B&B hotel, or any other type of homelessness service; sofa surfed; slept rough; applied to the council as homeless; none of the above.

Table S10: Percentage of people with self-identified alcohol problem – current or in recovery (n=537) and whether they receive adequate support.

	% (95% CI)		
	All who self-identified alcohol problem	Current alcohol problem	In recovery
All who self-identified alcohol problem	–	55.3 (51.1, 59.5)	44.7 (40.5, 48.9)
No support needed	26.8 (23.1, 30.6)	18.5 (14.1, 22.9)	37.1 (31.0, 43.2)
No support but would be helpful	16.2 (13.1, 19.3)	24.6 (19.7, 29.5)	5.8 (2.9, 8.8)
Support that meets needs	38.2 (34.1, 42.3)	30.0 (24.8, 35.2)	48.3 (42.0, 54.7)
Support but would like more	18.8 (15.5, 22.1)	26.9 (21.9, 32.0)	8.8 (5.2, 12.3)

Table S11: Type of support received by those with a self-identified alcohol problem who receive support for it (n=306; options not mutually exclusive).

**Type of support**	**% (95% CI)**
Advice and information	44.4 (38.9, 50.0)
Mutual aid	44.1 (38.6, 49.7)
Community prescribing	14.4 (10.4, 18.3)
Counselling or psychological support	28.8 (23.7, 33.8)
Day programme in community	21.2 (16.7, 25.8)
Detox	11.8 (8.2, 15.4)
Residential rehabilitation	7.5 (4.6, 10.5)
Aftercare	8.5 (5.4, 11.6)
Peer support	26.1 (21.2, 31.1)
Other support	17.3 (13.1, 21.6)

Table S12: Odds ratios for receiving different types of support by age, gender, and type of homelessness (n=306).

Characteristics		Information & peer-led support	Psychological & community-based interventions	Medical & residential treatment
Age (ref. ≥35)	OR	0.98 (0.55, 1.80)	1.21 (0.71, 2.05)	0.85 (0.44, 1.56)
	OR_{adj}	1.07 (0.59, 2.01)	1.22 (0.71, 2.11)	0.98 (0.50, 1.86)
Gender (ref. female)	OR	0.99 (0.54, 1.77)	0.80 (0.47, 1.35)	1.34 (0.72, 2.62)
	OR_{adj}	1.03 (0.55, 1.88)	0.83 (0.48, 1.43)	1.56 (0.81, 3.21)
Temporary or supported housing (ref. own tenancy)	OR	0.48 (0.14, 1.32)	1.20 (0.53, 2.80)	0.65 (0.27, 1.67)
	OR_{adj}	0.48 (0.13, 1.32)	1.16 (0.51, 2.73)	0.65 (0.27, 1.70)
Unsheltered or hidden homelessness (ref. own tenancy)	OR	0.35 (0.09, 1.18)	0.91 (0.32, 2.59)	0.67 (0.21, 2.14)
	OR_{adj}	0.34 (0.08, 1.18)	0.87 (0.30, 2.54)	0.64 (0.19, 2.11)
Other type (ref. own tenancy)	OR	0.55 (0.08, 4.69)	1.36 (0.27, 7.05)	3.75 (0.73, 22.50)
	OR_{adj}	0.56 (0.08, 4.88)	1.29 (0.25, 6.80)	4.53 (0.85, 28.39)
Number of different types of homelessness experienced[2]	OR	0.88 (0.67, 1.13)	1.34 (1.07, 1.70)	1.16 (0.89, 1.53)
	OR_{adj}	0.88 (0.67, 1.13)	1.37 (1.09, 1.74)	1.14 (0.87, 1.51)

Abbreviations: OR, odds ratio; OR_{adj}, adjusted odds ratio, with respective other variables used for adjustment. For number of types of homelessness experienced, adjusted for age and gender. [2]Options included: stayed at a hostel, foyer, refuge, night shelter or B&B hotel, or any other type of homelessness service; sofa surfed; slept rough; applied to the council as homeless; none of the above.

6. References

1. Ministry of Housing, Communities & Local Government. Homelessness data: notes and definitions. UK Government. 2 April, 2025. Updated 3 April 2018. Accessed 2 April, 2025. https://www.gov.uk/guidance/homelessness-data-notes-and-definitions
2. Bramley G. *Homelessness Monitor Research Programme: Technical report on updated baseline estimates and scenario projections 2023*. 2023. https://researchportal.hw.ac.uk/en/publications/homelessness-monitor-research-programme-technical-report-on-updat
3. Crisis. Homelessness in England. Crisis. Accessed 16 December, 2025. https://www.crisis.org.uk/ending-homelessness/homelessness-monitor/the-homelessness-monitor-england-2025/
4. van Buuren S. *Package 'mice'*. 2021. Accessed 17 July 2023. https://cran.r-project.org/web/packages/mice/mice.pdf
5. van Buuren S. *Multivariate Imputation by Chained Equations*. Vol. 3.16.0. 2023. Accessed 18 November 2024. https://cran.r-project.org/web/packages/mice/mice.pdf
6. Office for National Statistics. Families and households in the UK: 2022. Office for National Statistics. 8 October, 2025. Updated 18 May 2023. Accessed 8 October, 2025. https://www.ons.gov.uk/peoplepopulationandcommunity/birthsdeathsandmarriages/families/bulletins/familiesandhouseholds/2022
7. Office for National Statistics. Population estimates for the UK, England, Wales, Scotland, and Northern Ireland: mid-2022. Office for National Statistics. 8 October 2025, 2025. Updated 26 March 2024. Accessed 8 October, 2025. https://www.ons.gov.uk/peoplepopulationandcommunity/populationandmigration/populationestimates/bulletins/annualmidyearpopulationestimates/mid2022
8. Bramley G. *Research on core homelessness and homeless projections: Technical report on new baseline estimates and scenario projections*. 2021.
9. Office for National Statistics. National population projections: 2018-based. ONS. 10 July, 2026. Accessed 10 July, 2026. https://www.ons.gov.uk/peoplepopulationandcommunity/populationandmigration/populationprojections/bulletins/nationalpopulationprojections/2018based
10. Office for National Statistics. Household projections for England: 2018-based. ONS. 10 July, 2026. Updated 29 June 2020. Accessed 10 July, 2026. https://www.ons.gov.uk/peoplepopulationandcommunity/populationandmigration/populationprojections/bulletins/householdprojectionsforengland/2018based
11. Stevens JE, Shireman E, Steinley D, Piasecki TM, Vinson D, Sher KJ. Item responses in quantity-frequency questionnaires: implications for data generalizability. *Assessment*. Jul 2020;27(5):1029–1044. doi:10.1177/1073191119858398
